# A comparative analysis of suicide attempts in left-behind children and non-left-behind children in rural China

**DOI:** 10.1371/journal.pone.0178743

**Published:** 2017-06-08

**Authors:** Hongjuan Chang, Qiuge Yan, Lina Tang, Juan Huang, Yuqiao Ma, Xiaozhou Ye, Yizhen Yu

**Affiliations:** The Department of Child, Adolescence and Woman Health Care, School of Public Health, Tongji Medical College, Huazhong University of Science and Technology, Wuhan, Peoples Republic of China; Stellenbosch University, SOUTH AFRICA

## Abstract

To estimate the prevalence of suicide attempts and explore the shared and unique factors influencing suicide risk in left-behind children (LBC) and non-left-behind children (NLBC) in rural China, this study collected data using a multi-stage cluster random sampling method from 13,952 children including 6,034 LBC and 7,918 NLBC. Sociodemographic characteristics, suicide attempts, neglect and physical abuse, negative life events, and loneliness were measured by self-reported questionnaires. Data were analyzed using logistic regression models. Gender and mother's education level were unique influential factors for NLBC while family structure type was a unique influential factor for LBC. The study provides two novel findings regarding NLBC specifically: 1. Children with optimal family socioeconomic status are more likely to report suicide attempts (odds ratio OR = 1)than are those in the general children population, OR 0.52 (95% CI: 0.39–0.70), and 2. Children with higher mother’s education level are subject to higher suicide rates in high school, OR 1.67 (95% CI: 1.13–2.46), and post-secondary education, OR 2.14 (95% CI: 1.37–3.37). The unique characteristics of LBC and NLBC in China suggest that investigating risk factors and determining the factors that might be targeted in intervention programs are urgently needed currently.

## Introduction

Suicide is a global health problem and a major public health concern. It is among the top causes of mortality worldwide, especially among adolescents in the West and developing countries [1]. Since suicide is a potentially preventable public health issue, it is important to examine its immediate precursors, especially suicide attempts (SAs), which refer to direct efforts to intentionally end one's own life, to aid in the development of future public health interventions [2,3].

Adolescents’ suicidal behavior has been reported to be associated with genetic, psychological, social, and familial factors with particular risks related to childhood adversities [4]. Many studies have pointed out a strong and graded association between exposure to adverse childhood experiences and SAs during adolescence and adulthood [4–6], and the most concerning adversities for suicidal behavior are abuse and neglect [7,8]; in developing countries, large numbers of adolescence have been exposed to these [9,10]. Evidence suggests that those who perceived their parents as almost always emotionally neglectful had an increased risk of psychiatric disorder including SAs [11]. Other major psychosocial factors related to suicidal behavior included the experience of negative events during the past 12 months and feelings of loneliness. Research demonstrated that attempters had experienced a greater number of negative life events prior to their attempts [12]. Furthermore, there was a significant dose—response relationship between negative life events experienced within the last year and increased risk of SAs [13]. In addition to negative events, it may be useful to consider the role of loneliness in predicting suicide risk in adolescents. Looking at correlates of suicide risk in existing studies, we found that children and adolescents who experienced chronically high levels of loneliness were more likely to report suicidality [14,15].

Left-behind children (LBC) are those rural children under 18 years of age who are left at home when one or both parents migrate to an urban area for work [16]. Since China has recently experienced unprecedented urbanization and changes in the structure of society, foreshadowing a population of rural-to-urban migrants and the offspring they leave behind [17], several studies have focused on the problems of LBC in this area [8,18]. The literature in general have shown that LBC had more psychological problems than NLBC, including inadequate family bonding, emotional vulnerability, and exposure to violence [8,19].

However, to date, it remains unclear whether the rate of SAs is higher in LBC than in NLBC in rural China among nationally representative samples. Nor has any research examined the specific difference of SA risk factors between LBC and NLBC within the current social context of China.

### Analyses

SPSS (version 17.0) was used for data analysis. Chi-squared tests were used to compare the difference of enumeration data variables across LBC and NLBC groups. Logistic regression analysis was performed to calculate the odds ratios (OR) and 95% confidence intervals (95% CI) for the factors related to SAs. All of the factors were chosen as the independent variables. All tests were two-tailed, and a *P*-value smaller than 0.05 was considered statistically significant.

## Materials and methods

### Participants and procedures

The data of the study were part of a nationwide study on mental health outcomes among adolescents in rural China, which was initiated in 2015. A multi-stage cluster random sampling method was adopted to collect data to represent all students from 7^th^ to 12^th^ grade in rural China. At the first stage of sampling, five districts were selected from the north, south, east, west, and middle part of China to represent the whole rural area of China. In the second stage, three counties or cities were chosen randomly in each province. In the last stage, schools were selected basing on their reported enrollment size. Excluding those who refused to participate in the study and who were absent from school, a total of 15,600 students were recruited in our study, and a consent letter was sent to their parents or guardians. Among the respondents, 1,648 were excluded due to incomplete questionnaires. Finally, 13,952 students were included in our analysis: 6,034 LBC and 7,918 NLBC with a mean age of 15.22 (SD = 1.81) years, ranging from 10 to 18 years. The actual response rate of the participants was 89.43% (13,952/15,600). Informed consent was obtained from the parents or guardians of each participant.

Data were collected by a group of trained postgraduate students, who explained the purpose and procedures of the study to participants. The students were instructed to place not their names but their student numbers on the questionnaires and to answer all the questions honestly. The students were also informed that their participation was voluntary and that the questionnaires did not represent a test as there were no correct or incorrect answers. We told each participant that they were to place the questionnaires in envelopes after completion and were not to hand them directly to school teachers or school personnel, and we promised them that the data would be used for scientific research only. The same written announcements were printed on the front of the questionnaires. The ethical protocol, including the questionnaires, was approved by the targeted schools and the Medical Ethics Committee of Tongji Medical College, Huazhong University of Science and Technology. Informed written consent was obtained from the parents or guardians of each participant.

### Measures

#### Sociodemographic characteristics

Information about gender, age, class, grade, school, and family structure type as well as education levels of parents and caregivers, family's socioeconomic status, parenting style, and family history of mental illness was collected.

#### Suicide attempts

Information on SAs was collected by the question, “During the preceding 12 months, how many times did you actually attempt suicide?” For this item, responses fall into two categories: “never” and “more than once.” The test—retest reliability of the question over two weeks was 99.41% in the present study.

#### Neglect and physical abuse

The prevalence of neglect and physical abuse was measured by a validated Chinese version of the Parents—Child Conflict Tactics Scale (CTSPC), which was developed to assess the subjective feelings of neglect and physical abuse among children or adolescents [20]. The scale consists of 17 items on four factors: neglect (e.g., ‘‘parents left you alone when you were in need of their company”), corporal punishment (e.g., ‘‘parents slapped you on the hand, arm, or leg”), physical maltreatment (e.g., ‘‘parents hit you with a fist or kicked you hard”), and severe physical maltreatment (e.g., ‘‘parents grabbed you around the neck and choked you”). The students in our study were asked how frequently they had encountered the listed behaviors in the past year using a three-point Likert scale covering none, once, and twice or more. Their responses were classified as “neglect or maltreatment of a particular type” if they had experienced one or more of the listed behaviors within the corresponding subscale. The neglect subscale consists of five items that range from 0 to 10. We defined scores of 0, 1–3, 4–6, and 7–10 as no, mild, moderate, and severe neglect, respectively. We defined the participants who did not experience the three subscales of physical abuse as having no physical abuse. Children who experienced corporal punishment only were defined as having mild physical abuse. Children who experienced physical maltreatment were defined as having moderate physical abuse, and children who experienced severe physical maltreatment were defined as having severe physical abuse. The internal consistency score of the whole scale was 0.86.

#### Negative life events

The Adolescent Self-Rating Life Events Checklist (ASRLEC), a 5-point Likert scale, aims to assess whether certain life events occurred to the participant as well as the effects, if any, in the past 12 months [21]. The scale consists of 27 items on six factors: interpersonal relationship (e.g., “I argued with my classmates”), study pressure (e.g., “I failed an examination”), being punished (e.g., “I was criticized and punished”), bereavement (e.g., “A family member/close friend died”), change for adaptation (e.g., “My living habits changed”), and others. Responses fall into five levels from 1 point (not at all) to 5 points (very much), and the higher the score, the greater their life pressure. At present, the scale is generally used to measure stress levels of Chinese students. For the present study, the Cronbach’s α of this scale was 0.92. The total scores were categorized into high, medium, and low negative life events groups using a cut-off of 1 standard deviation (SD) above, between, and below the mean.

#### Loneliness

Loneliness was measured by the University of California Los Angeles Loneliness Scale (UCLA LS) [22], a unidimensional, self-report measure of perceived isolation (e.g., items such as “I often feel that there is nobody who cares about me.”). Items are rated on 5-point scale with higher scores indicating greater loneliness. The UCLA LS shows high internal consistency and adequate convergent validity [23]. Internal consistency in our study was high for the sample (Cronbach’s α = 0.79). The score hierarchies were in accordance with the negative life events scale.

## Results

### Demographic characteristics

The demographic characteristics of the LBC and NLBC groups are shown in Table 1. From the total sample of 13,952, the NLBC group represented 56.85% (n = 7,918), and the LBC group comprised 43.25% (n = 6,034). In 23.36% of cases (n = 3,257), the father had migrated to an urban area for work; in 3.14% (n = 438) it was the mother, and in 16.72% (n = 3,257) of cases, both parents had migrated. Most students in the sample were taken care of by the mother on a daily basis (68.02%). Most students were from a middle socioeconomic background (54.97%). A small proportion of the children were only children (34.23%). There was no significant difference between the LBC and non-LBC groups in terms of gender. However, we observed large differences between the two groups. Specifically, the LBC group had a mean socioeconomic status that was 1.55% higher than the non-LBC group, the education level of mothers in the LBC group was 11.62% higher, the number of cross-generational families was 5.79% higher, and the rate of being an only child was 14.72% higher. Conversely, a democratic parenting style was 3.39% higher in the non-LBC group, and family history of mental illness was 1.12% higher. Generally, positive factors were higher in the NLBC group than in the LBC group, and vice versa.

**Table 1:** The demographic characteristics of the LBC and NLBC groups.

Variables	Total sample (n = 13952)		NLBC(n = 7918)		LBC(n = 6034)		χ2	*p*
	n	Constituent Ratio (%)	n	Constituent Ratio (%)	n	Constituent Ratio (%)		
**Gender**							1.9	0.17
Boy	7345	52.76	4125	52.25	3220	53.43		
Girl	6577	47.24	3770	47.75	2807	46.57		
**Age**							**47.14**	**<0.01**
10–12	700	5.02	447	5.65	253	4.19		
13–15	6988	50.1	4100	51.8	2888	47.88		
16–18	6259	44.88	3368	42.55	2891	47.93		
**Grade**							**79.24**	**<0.01**
7	2556	18.32	1519	19.18	1037	17.19		
8	2678	19.19	1639	20.7	1039	17.22		
9	2335	16.74	1388	17.53	947	15.69		
10	2254	16.16	1196	15.1	1058	17.53		
11	2176	15.6	1175	14.84	1001	16.59		
12	1953	14	1001	12.64	952	15.78		
**Only child**							**327.61**	**<0.01**
Yes	4751	34.23	3197	40.6	1554	25.88		
No	9128	65.77	4678	59.4	4450	74.12		
**Family structure types**							**430.96**	**<0.01**
Nuclear families	8925	64.72	5454	69.3	3471	58.62		
Extended families	3477	25.21	1853	23.55	1624	27.43		
Single parent family	672	4.87	341	4.33	331	5.59		
Remarried families	257	1.86	155	1.97	102	1.72		
Cross-Generation Family	460	3.34	67	0.85	393	6.64		
**Father's education level**							**501.61**	**<0.01**
Primary School or Below	2733	19.86	1338	17.09	1395	23.52		
Junior middle school	7473	54.3	3979	50.81	3494	58.9		
High school or technical secondary school	2504	18.19	1625	20.75	879	14.82		
College or above	1053	7.65	889	11.35	164	2.76		
**Mother's education level**							**740.16**	**<0.01**
Primary School or Below	4739	34.46	2118	27.08	2621	44.18		
Junior middle school	6447	46.88	3735	47.76	2712	45.72		
High school or technical secondary school	1835	13.34	1334	17.06	501	8.45		
College or above	731	5.32	633	8.09	98	1.65		
**Identity of primary caregiver**								
Mother	9372	68.02	6038	77.8	3334	55.41	**1480.71**	**<0.01**
Father	1197	8.69	787	10.14	410	6.81		
Grandparents	1855	13.46	369	4.75	1486	24.7		
Brothers or sisters	68	0.49	20	0.26	48	0.8		
Other relatives	248	1.8	48	0.62	200	3.32		
Oneself	1038	7.53	499	6.43	539	8.96		
**Family's socioeconomic status**	4330	32.65	2444	32.66	1886	32.64	**7.71**	**0.02**
Optimal								
General	7291	54.97	4164	55.64	3127	54.11		
Poor	1642	12.38	876	11.7	766	13.25		
**Caregiver's education level**	3883	28.38	1501	19.42	**2382**	**40**	**847.98**	**<0.01**
Primary School or Below								
Junior middle school	6085	44.47	3669	47.47	**2416**	**40.57**		
High school or technical secondary school	2845	20.79	1846	23.88	999	16.78		
College or above	871	6.37	713	9.22	158	2.65		
**Parenting style**	11253	84.45	6486	85.92	4767	82.53	**56.46**	**<0.01**
Authoritative								
Permissive	501	3.76	237	3.14	264	4.57		
Neglectful	217	1.63	85	1.13	132	2.29		
Authoritarian	84	0.63	36	0.48	48	**0.83**		
Constantly changing	1270	9.53	705	9.34	565	**9.78**		
**Family history of mental illness**								
Yes	543	4.15	271	3.67	272	4.79	**10.11**	**<0.01**
No	12528	95.85	7118	96.33	5410	95.21		

Note. *P*: Difference significance test of the constituent ratio of within the LBC group and NLBC group

Percentages of variables may not add up to 100% due to missing

### Association of demographic characteristics with suicide attempts

The self-reported one-year prevalence rate of SAs was 3.24%. Across the whole sample, girls were more likely to have attempted to commit suicide than were boys, OR 1.22 (95%CI: 1.01–1.47). However, in the LBC group, this difference was not significant. Additionally, significant differences were found between boys in the LBC and non-LBC groups (*P* < 0.01) in terms of one-year prevalence of SAs. Parenting style was significantly associated with SAs. This association was further replicated in multiple regression analysis when controlling for age, gender, abuse, and neglect. Additionally, family history of mental illness was associated with an increase in the risk of SAs, not only in the LBC group, OR 0.33 (95%CI: 0.22–0.51), but also in the non-LBC group, OR 0.37 (95%CI: 0.23–0.60) (shown in Table 2).

**Table 2:** Association of demographic characteristics with suicide attempts.

Variables	Total sample		NLBC		LBC		χ2	*p*
	Prevalence Rate (%)	OR(95%CI)	Prevalence Rate (%)	OR(95%CI)	Prevalence Rate (%)	OR(95%CI)		
**Total sample**	3.24		2.86		3.75		**8.58**	**<0.01**
**Gender**								
Boy	2.95	1.00	2.39	1.00	3.67	1.00	**10.22**	**<0.01**
Girl	3.56	1.22(1.01–1.47)*	3.36	1.42(1.09–1.86)**	3.82	1.04(0.80–1.36)	0.96	0.34
**Age**								
10–12	2.92	1.00	2.96	1.00	2.83	1.00	0.01	1.00
13–15	3.97	1.38(0.87–2.18)	3.50	1.19(0.67–2.12)	4.64	1.67(0.77–3.59)	**5.73**	**0.02**
16–18	2.53	0.87(0.53–1.36)	2.15	0.72(0.38–1.27)	2.99	1.06(0.47–2.26)	**4.22**	**0.05**
**Grade**								
7	3.62	1.00	3.35	1.00	4.02	1.00	0.76	0.39
8	4.03	1.12(0.84–1.49)	3.32	0.99(0.67–1.47)	5.16	1.30(0.86–1.97)	**5.49**	**0.03**
9	4.13	1.15(0.85–1.54)	3.83	1.15(0.77–1.70)	4.58	1.15(0.74–1.77)	0.77	0.39
10	3.48	0.96(0.70–1.31)	2.82	0.84(0.53–1.31)	4.22	1.05(0.68–1.62)	3.25	0.08
11	2.27	0.62(0.43–0.88)**	2.31	0.68(0.41–1.10)	2.21	0.54(0.32–0.91)*	0.03	0.89
12	1.45	0.39(0.26–0.60)**	0.71	0.21(0.09–0.46)**	2.23	0.55(0.32–0.93)*	**7.83**	**0.01**
**Only child**								
Yes	3.16	1.00	2.60	1.00	4.32	1.00	**9.97**	**<0.01**
No	3.26	1.03(0.85–1.26)	2.96	1.14(0.87–1.51)	3.58	0.82(0.61–1.10)	2.78	0.10
**Family structure types**								
Nuclear families	3.00	1.00	2.81	1.00	3.30	1.00	1.76	0.20
Extended families	3.28	1.10(0.88–1.37)	2.91	1.04(0.76–1.43)	3.69	1.12(0.81–1.55)	1.66	0.21
Single parent family	3.75	1.26(0.83–1.92)	2.68	0.95(0.48–1.88)	4.85	1.49(0.88–2.56)	2.17	0.16
Remarried families	6.32	2.18(1.30–3.67)**	2.65	0.94(0.34–2.58)	11.76	3.90(2.10–7.41)**	**8.54**	**0.01**
Cross-Generation Family	4.85	1.65(1.05–2.57)*	6.06	2.23(0.80–6.22)	4.64	1.42(0.86–2.37)	0.25	0.54
**Father's education level**								
Primary School or Below	3.03	1.00	2.35	1.00	3.68	1.00	4.05	0.06
Junior middle school	3.03	1.00(0.77–1.29)	2.56	1.09(0.72–1.64)	3.57	0.97(0.70–1.35)	**6.40**	**0.01**
High school or technical secondary school	3.81	1.23(0.94–1.71)	3.49	1.50(0.96–2.33)	4.43	1.21(0.79–1.87)	1.38	0.27
College or above	4.23	1.41(0.97–2.05)	4.09	1.77(1.09–2.88)*	5.03	1.39(0.65–2.97)	0.30	0.53
**Mother's education level**								
Primary School or Below	3.06	1.00	2.45	1.00	3.55	1.00	**4.74**	**0.03**
Junior middle school	2.96	0.97(0.77–1.20)	2.36	0.96(0.68–1.37)	3.78	1.07(0.80–1.42)	**10.79**	**<0.01**
High school or technical secondary school	4.21	1.39(1.05–1.85)*	4.02	1.67(1.13–2.46)**	4.72	1.35(0.84–2.15)	0.44	0.51
College or above	5.12	1.71(1.18–2.47)**	5.11	2.14(1.37–3.37)**	5.15	1.47(0.59–3.71)	0.00	1.00
**Identity of main caregiver**								
Mother	2.92	1.00	2.67	1.00	3.38	1.00	**3.78**	**0.05**
Father	3.42	1.18(0.84–1.65)	3.13	1.18(0.76–1.82)	3.96	1.18(0.69–2.01)	0.55	0.50
Grandparents	4.16	1.44(1.11–1.87)**	4.70	1.80(1.08–3.00)*	4.02	1.20(0.87–1.65)	0.33	0.56
Brothers or sisters	5.97	2.11(0.76–5.84)	10.00	4.05(0.93–17.61)	4.26	1.27(0.30–5.29)	0.83	0.58
Other relatives	7.44	2.67(1.63–4.38)**	6.38	2.49(0.76–8.09)	7.69	2.38(1.36–4.16)**	0.09	1.00
Oneself	3.59	1.24(0.87–1.76)	3.41	1.29(0.78–2.14)	3.76	1.12(0.69–181)	0.09	0.87
**Family's socioeconomic status**								
Optimal	4.14	1.00	4.02	1.00	4.30	1.00	0.21	0.70
General	2.67	0.64(0.52–0.78)**	2.14	0.52(0.39–0.70)**	3.37	0.79(0.58–1.05)	**10.32**	**<0.01**
Poor	3.67	0.88(0.65–1.19)	3.28	0.81(0.53–1.24)	4.12	0.97(0.63–1.46)	0.80	0.43
**Caregiver's education level**								
Primary School or Below	2.95	1.00	2.58	1.00	3.19	1.00	1.15	0.33
Junior middle school	3.19	1.08(0.85–1.37)	2.60	1.01(0.69–1.47)	4.08	1.29(0.95–1.76)	**10.17**	**<0.01**
High school or technical secondary school	3.09	1.05(0.79–1.39)	2.79	1.08(0.71–1.66)	3.67	1.16(0.77–1.73)	1.66	0.21
College or above	5.83	2.03(1.45–2.86)**	5.40	2.15(1.36–3.41)**	7.79	2.57(1.36–4.83)**	1.32	0.26
**Parenting style**								
Authoritative	2.67	1.00	2.39	1.00	3.04	1.00	**4.46**	**0.04**
Permissive	2.64	0.99(0.56–1.74)	2.15	0.90(0.36–2.20)	3.09	1.02(0.49–2.10)	0.42	0.58
Neglectful	7.94	3.15(1.89–5.24)**	7.14	3.14(1.35–7.32)**	8.46	2.95(1.55–5.58)**	0.12	0.80
Authoritarian	16.87	7.41(4.12–13.31)**	20.00	10.21(4.39–23.74)**	14.58	5.44(2.40–12.33)**	0.42	0.56
Constantly changing	6.84	2.68(2.09–3.49)**	5.83	2.53(1.77–3.62)**	8.09	2.81(1.99–3.98)**	2.47	0.14
**Family history of mental illness**								
yes	8.43	1.00	7.09	1.00	9.77	1.00	1.25	0.28
No	3.05	0.34(0.25–0.47)**	2.72	0.37(0.23–0.60)**	3.49	0.33(0.22–0.51)**	**5.96**	**0.02**

Note. *P*: Difference significance test of the prevalence rate of suicide attempts between subgroups in the LBC and NLBC group

* *P* < 0.05;

** *P* < 0.01

Several variables in the subgroup analyses were not significantly different (*P* > 0.05) across groups, comprising female gender, age between 10 and 12 years old, not only child status, nuclear families, extended families, single-parent families, parents and caregivers with a college or high school degree, and family history of mental illness. However, there were differences between the LBC and non-LBC groups, including the number of children in 12th grade, male gender, no family history of mental illness, parents and caregivers who completed junior middle school, and only child status.

### Association of CTSPC, ASRLEC, and UCLA LS scales with suicide attempts

Importantly, 66.97% of the children in rural China had experienced neglect. Nearly half of the students (46.77%) had experienced abuse categorized as mild, moderate, or severe. In total, 72.66% of children reported having a “negative life” at a level classified as moderate or above, and 69.90% said that they had experienced loneliness, again to a degree categorized as moderate or above. The proportion of LBC experiencing a high level of loneliness was 3.75%, higher than the non-LBC group (χ^2^ = 11.58, *P* < 0.01). Overall, the three scales were significantly positively associated with SAs. Considering the difference between the LBC and non-LBC groups, the prevalence of SAs was higher in the LBC group than in the non-LBC group in terms of those children scoring on the middle level of the abuse scale (χ^2^ = 5.55, *P* < 0.05) and high in terms of loneliness (χ^2^ = 11.90, *P* < 0.05). There were no significant differences between the LBC and non-LBC groups at other levels (shown in Table 3).

**Table 3:** Association of CTSPC, ASRLEC, and UCLA LS scales with suicide attempt.

Variables	Total sample		non-LBC		LBC		χ2	*p*
	Constituent ratio (%)	OR(95%CI)	Constituent ratio (%)	OR(95%CI)	Constituent ratio (%)	OR(95%CI)		
**Abuse**								
No	53.23	1	56.16	1	49.39	1	0.89	0.39
Mild	15.68	1.23(0.89–1.70)	14.80	1.30(0.83–2.02)	16.84	1.13(0.70–1.83)	0.01	1
Moderate	27.08	2.53(2.02–3.17)**	25.35	2.24(1.63–3.08)**	29.34	2.75(1.99–3.81)**	5.55	**0.02**
Severe	4	8.27(6.17–11.09)**	3.68	8.58(5.73–12.86)**	4.42	7.79(5.08–11.91)**	0.07	0.81
**Neglect**								
No	33.07	1	38.08	1	26.47	1	0.96	0.37
Mild	37.98	1.75(1.30–2.36)**	36.66	1.87(1.26–2.76)**	39.71	1.55(0.98–2.45)	0.12	0.72
Moderate	22.55	3.63(2.72–4.85)**	20.10	3.68(2.49–5.42)**	25.78	3.36(2.16–5.21)**	0.89	0.37
Severe	6.4	6.60(4.73–9.22)**	5.16	6.75(4.22–10.81)**	8.04	5.98(3.67–9.76)**	0.27	0.64
**Loneliness**								
Low	13.81	1	15.18	1	12.01	1	1.44	0.23
Medium	69.9	1.60(1.08–2.36)*	70.20	1.99(1.16–3.41)*	69.51	1.19(0.67–2.09)	0.24	0.64
High	16.29	5.09(3.41–7.59)**	14.63	4.50(2.54–7.95)**	18.48	5.10(2.88–8.98)**	**11.9**	**<0.01**
**Negative events**								
Low	13.34	1	15.92	1	9.97	1	2.02	0.16
Medium	72.66	3.21(1.87–5.52)**	71.76	4.18(1.95–8.97)**	73.85	2.18(1.00–4.67)*	0.52	0.47
High	13.99	11.75(6.77–20.37)**	12.33	14.57(6.68–31.81)**	16.18	8.21(3.76–17.81)**	1.09	0.32

Note. *P*: Difference significance test of the constituent ratio of subgroups in the LBC group and non-LBC group;

* *P* < 0.05;

** *P* < 0.01

### Association of LBC characteristics with suicide attempts

The risk of SA in children for whom both parents had migrated was significantly higher than in children for whom either the mother or the father had migrated for work, OR 1.39 (95%CI: 1.02–1.78). Moreover, the risk of SAs was significantly higher in children who communicated with their parent(s) every six months or more compared with those who communicated with a frequency of more than once per week, OR 2.39 (95%CI: 1.59–4.36). For children whose parents had migrated to urban areas for work when they were between 7 and 12 years old, the risk of SAs was reduced compared with those whose parents had left when they were under 2 years old, OR 0.62 (95%CI: 0.43–0.89). However, there were no significant differences across groups in terms of length of time since parents had migrated, frequency of reunions with parents, and length of parents’ stay at home during each reunion (shown in Table 4).

**Table 4:** Association of LBC characteristic information and suicide attempts.

Variables	Constituent ratio (%)	Prevalence rate (%)	OR(95%CI)	*p*
**Identity of who migrate to urban area for work**				
Father	54.05	3.27	1	
Mother	7.27	4.17	1.25(0.77–2.14)	0.34
Both of father and mother	38.68	4.36	**1.39(1.02–1.78)**	**0.04**
**Length of parent(s) migrate to work**				
<1 year	35.49	3.62	1	
2–5 year	19.06	3.5	0.97(0.65–1.44)	0.87
6–10 year	17.15	4.19	1.17(0.79–1.72)	0.44
More than 10 year	28.31	4.03	1.12(0.80–1.57)	0.51
**Age of you when your parents migrate to work**				
<2 year	21.66	4.91	1	
3–6 year	27.92	3.75	0.76(0.53–1.09)	0.13
7–12 year	31.77	3.08	**0.62(0.43–0.89)**	**0.01**
>13 year	18.65	3.88	0.78(0.52–1.16)	0.22
**Frequency of communication with parents**				
<1 week	60.27	3.36	1	
2 week	21.47	3.71	1.12(0.79–1.56)	0.56
1 month	13.09	4.06	1.21(0.82–1.82)	0.34
6 month and more	3.89	7.67	**2.39(1.59–4.36)**	**<0.01**
**Frequency of reunions with parents**				
<1 week	12.76	4.15	1	
2 week	6.1	3.94	0.95(0.50–1.80)	0.87
1 month	17.9	3.07	0.73(0.44–1.21)	0.22
6 month	48.63	3.55	0.85(0.56–1.28)	0.44
1 year and more	14.6	4.81	1.17(0.72–1.88)	0.52
**Length of parents’ stay at home during each reunion**				
<1 week	41.03	3.71	1	
<1 month	38.41	3.82	1.03(0.75–1.41)	0.85
1–3 month	13	3.65	0.98(0.63–1.54)	0.94
3 month or more	7.56	2.91	0.78(0.42–1.45)	0.42

### Logistic regression of potential predictors of suicide attempts

Multivariate logistic regression was performed to examine the influence of certain predictors on SAs (as summarized in Table 5). After adjusting for socioeconomic and demographic characteristics, neglect, physical abuse, negative life events, and loneliness were strongly associated with risk of SAs in both LBC and NLBC groups. Gender was a significant variable in the single factor analysis but did not enter the regression equation in the total sample. Whereas gender was a significant predictor in the NLBC group, OR 1.43 (95%CI: 1.17–2.16), it was not in the LBC group. Conversely, whether either parent had remarried was an important predictor in the LBC group but not in the NLBC group, OR 3.55 (95%CI: 1.49–6.57).

**Table 5:** Results of logistic regression analyses predicting suicide attempts of NLBC and LBC groups.

Variables	model I (total sample, Nagelkerke R^2^ = 0.13)				modelII (NLBC, Nagelkerke R^2^ = 0.13)				modelIII (LBC, Nagelkerke R^2^ = 0.14)			
	*B*	*S*.*E*.	*P*	OR(95%CI)	*B*	*S*.*E*.	*P*	*OR*(95%CI)	*B*	*S*.*E*.	*P*	*OR*(95%CI)
**Constant**	-3.9	0.73	**<0.01**	**0.01**	-5.8	0.87	**<0.01**	**<0.01**	-5.4	0.84	**<0.01**	
**Gender** (Boy = 1)					0.36	0.16	**0.03**	**1.43(1.17–2.16)**				
**Age**(10 = 1)	-0.1	0.03	**<0.01**	**0.88(0.82–0.93)**	-0.1	0.05	**0.01**	**0.88(0.81–0.97)**	-0.1	0.05	**0.01**	**0.88(0.72–1.50)**
**Family structure types** (NuclearFamilies = 1)											**0.02**	
Extended families									0.02	0.19	0.91	1.02(0.74–1.65)
Single parent family									0.21	0.33	0.52	1.24(0.91–2.47)
Remarried families									1.27	0.39	**<0.01**	3.55(1.49–6.57)
Cross-generation Family									0.22	0.31	0.47	1.25(0.75–2.34)
**Mother's education level** (Primary school or below = 1)			**<0.01**				**<0.01**					
Junior middle school	0.03	0.13	0.84	1.03(0.80–1.34)	-0	0.21	0.85	0.96(0.66–1.42)				
High school or technical secondary school	0.47	0.17	**0.01**	**1.59(1.15–2.21)**	0.5	0.23	**0.03**	**1.64(1.07–2.55)**				
College or above	0.76	0.21	**<0.01**	**2.14(1.41–3.27)**	0.95	0.26	**<0.01**	**2.58(1.54–4.10)**				
**Family's socioeconomic Status** (Optimal = 1)	-0.2	0.09	**0.02**	**0.82(0.69–0.97)**								
**Parenting style** (Authoritative = 1)			**<0.01**				**0.01**				**0.02**	
Permissive	-0.3	0.32	0.44	0.78(0.41–1.46)	-0.1	0.48	0.88	0.92(0.32–2.05)	-0.3	0.43	0.53	.76(0.31–1.65)
Neglectful	0.73	0.3	**0.02**	**2.08(1.22–3.85)**	1.01	0.5	**0.04**	**2.75(0.88–6.15)**	0.69	0.38	0.07	1.99(0.91–4.04)
Authoritarian	1.21	0.37	**<0.01**	**3.34(1.60–6.68)**	1.35	0.55	**0.01**	**3.86(1.30–10.96)**	1.08	0.5	**0.03**	**2.95(1.26–7.90)**
Constantly changing	0.49	0.15	**<0.01**	**1.63(1.24–2.21)**	0.43	0.21	**0.04**	**1.54(1.18–1.61)**	0.5	0.21	**0.02**	**1.64(1.12–2.49)**
**Family history of mental illness** (No = 1)	0.55	0.21	**0.01**	**1.76(1.19–2.63)**								
**Abuse** (No = 1)	0.26	0.06	**<0.01**	**1.30(1.15–1.45)**	0.31	0.08	**<0.01**	**1.37(1.18–1.61)**	0.25	0.09	**<0.01**	**1.28(1.12–1.55)**
**Neglect** (No = 1)	0.31	0.07	**<0.01**	**1.37(1.22–1.58)**	0.29	0.09	**<0.01**	**1.34(1.15–1.62)**	0.29	0.1	**<0.01**	**1.33(1.13–1.62)**
**Loneliness(Low = 1)**	0.53	0.11	**<0.01**	**1.70(1.40–2.12)**	0.39	0.15	**0.01**	**1.48(1.09–1.91)**	0.66	0.15	**<0.01**	**1.94(1.50–2.67)**
**Negative events** (Low = 1)	0.85	0.11	**<0.01**	**2.34(1.88–2.92)**	0.9	0.16	**<0.01**	**2.46(1.86–3.37)**	0.81	0.16	**<0.01**	**2.26(1.57–2.91)**

Note. B: Unstandardized Coefficients; S.E.: Standard Error

Overall, the higher the family’s socioeconomic status, the greater the risk of suicide, OR 0.82 (95%CI: 0.69–0.97), but this was not found in either subgroup on its own. Family history of mental illness was a predictor within the total sample, OR 1.72 (95%CI: 1.1–92.63) but, again, was not found uniquely in either subgroup. All the equations indicated that risk of SAs lowered as age increased.

## Discussion

This quantitative survey-based study presents cross-sectional evidence suggesting that LBC are disadvantaged in terms of their families’ socioeconomic situations and parenting styles. As described in previous literature, parental migration from rural to urban areas often leads to a higher income and enhanced socioeconomic status [24]. However, reduced parental care in the absence of one or both parents negatively influences a child’s development and probably also harms a child’s psychological and physical health due to lessened family control and supervision and weakened parental support and guidance combined with undermined parent—child bonding. This combination of adversities could feasibly manifest in a higher proportion of children being left behind. Given the differences between LBC and NLBC in demographic characteristics, it is not difficult to explain why prevalence of SAs was higher in the LBC group than in the NLBC group.

Prevalence of SAs in rural China was 3.24%, far less than estimates reported in Western societies [25,26] but similar to reports in China [27]. The increased likelihood of girls having attempted suicide relative to boys is consistent with many previous studies [28,29]. Previous research has suggested that girls are more sensitive to interpersonal relationships, including those with peers and family. They were also more inclined than boys to hide negative emotions [30]. The huge difference in suicidal behaviors between urban and rural areas is an important characteristic in China [31]. However, few differences were found between urban and rural areas in the present study (3.5% vs. 3.24%)—a departure from what we found previously [27]. When interpreting these estimates of SAs prevalence, it is important to note that there may be unaccounted-for factors that could cause inaccurate self-reporting. In this study, we asked the students to write their student numbers on the questionnaires so that they could be identified for further research. It is possible that the students may have been hesitant or reluctant to give information on a written survey pertaining to such a sensitive topic as suicidal behavior. Additionally, social desirability bias should be considered. With these limitations in mind, the prevalence of suicide in rural China, particularly in LBC, still deserves serious attention.

Interestingly, in single factor analysis, the higher the mother’s education level, the higher a child’s suicide rate in high school or technical secondary school as well as in college or higher education. Moreover, the prevalence of SAs was highest in children whose caregivers had been educated to a college degree or above. Similar associations were not found in relation to the father’s level of education. This was found across both groups of children, a finding that has not been commonly reported. The critical emphasis on education is a unique characteristic of the Chinese culture [29,32,33]. The putative explanation is that highly educated parents will have high expectations of their children, which, in turn, could exacerbate academic pressure. The novel findings suggest that further studies should be conducted to help us understand the relationship between academic stress and risk behavior, indexed by SAs, in China. It is important to note that, the conclusion requires further validation because these data are based on retrospective self-reports of the occurrence of SAs rather than actual attempt, which introduces potential problems with reporting bias leading to false associations. Due to reporting bias, it is difficult to know whether these reported suicide attempts are really correlated with actual suicides. It is possible that those with better family background report more suicide attempts whereas those with worse family background are more likely to commit suicide, which needs further research and demonstration. In addition, the reliability of the inference may be limited according to the small sample size of subgroup.

Despite differences in the demographics of our LBC and NLBC, negative parenting styles lead to SAs in both groups. This finding highlights the salient role of parenting style, and previous empirical evidence can shed light on this matter [34]. Furthermore, there is an accumulating body of literature suggesting that children with remarried parents are at increased risk of self-harm and SAs. These children must face a more complicated living situation, requiring communication with their original family as well as step families [29,35]. Our findings support the idea that LBC from remarried families were more at risk for attempting suicide. However, NLBC from cross-generational families were more at risk for attempting suicide.

Compared with Western counterparts, Chinese children endure more neglect and physical abuse. In our study, the prevalence of neglect and physical abuse was 66.97% and 46.77%, respectively. However, it was estimated that the prevalence of physical abuse in Australian children ranges from 5% to 18% whereas neglect ranges from 2% to 12% [36]. Our findings show that neglect and physical abuse is also an important issue in rural China. In the current study, neglect and physical abuse were both risk factors for SAs in both the total sample and subgroups. Previous research has found that adolescents who experience neglect and physical abuse may be at risk for SAs [37]. Indeed, not all LBC are necessarily neglected, but parents should communicate with their children more frequently. In the current study, we found that higher communication frequency between parents and children was associated with lower SAs.

The strongest predictor of SAs among the three scales was negative life events. This conclusion was consistent with evidence of a correlation between negative life events in the preceding year and SAs [38]. In this vein, it is likely that children already exposed to negative life events such as failing a test, stress from interpersonal relationships, and pressure to enter a better high school or college should receive timely psychological support and encouragement from their parents or guardians. Researchers found that resilience could be promoted through parental monitoring, disclosure, social support, and family-based interventions [39]. Further research is required to identify protective factors against students’ suicidality within rural China.

The present study indicates that loneliness was the most common and important experience of both groups. Children who experienced high levels of loneliness had higher levels of SAs in the LBC group than in the NLBC group. This is an important finding. In the present study, we found that the risk of SAs in children who had been left behind by both parents was significantly lower than in children for whom only one parent had left. Parents who leave home should be aware of the prominence of their involvement in their children’s lives even if they cannot physically accompany them. They should communicate closely with their children via telecommunication to reduce the loneliness of LBC.

In this comparative study, compared with families with an optimal socioeconomic status, the general group had a lower risk of SAs. This was only found in the NLBC and not in LBC. Nonetheless, there were no statistical differences between the optimal and low socioeconomic status groups. In contrast, according to the existing literature, family economic adversity significantly affects adolescents’ suicidal behaviors [29,40]. Parents in families with a high socioeconomic status may work more often than those from general levels, which may lead to neglect or a spoiled parenting style in the context of rural China. If this conjecture is correct, we must study the relationship between family economic status and neglect of children in rural China. Furthermore, we identified age as a protective factor; older LBCs had a smaller risk for SAs.

Our novel findings suggest that more comparative studies are required to explore the role of parental migration to urban areas for work in negative outcomes in LBC. The unique characteristics of LBC and NLBC in China suggest a pressing need to investigate risk factors and to determine the factors that might be targeted in intervention programs. This study is relevant to future international studies as well as informing culturally based prevention and intervention programs and services targeted toward children in rural China.

This study should be interpreted in light of certain limitations. First, these data are based on retrospective self-reports of the occurrence of SAs rather than actual attempt, which introduces potential problems with reporting bias leading to false associations, so the conclusion requires further validation. Second, although the study achieved a relatively large sample size, there were some demographic differences between the LBC and NLBC, which could indicate selection bias. The current study was a school-based sample, and LBC may drop out of school before finishing compulsory education and before their NLBC counterparts [41]. Further studies should incorporate a group of children no longer attending school to minimize this bias. Third, these data are based on retrospective self-reported data of the occurrence of suicidal behaviors in the past 12 months, which may be underestimated because of recall bias, introducing potential problems with under-reporting in terms of pseudo-anonymity and socioeconomic background. Finally, this study was cross-sectional in nature, meaning that no causal inferences can be made. Thus, more longitudinal research is needed to understand the mechanism of how risk factors lead to SAs on individual and group levels.

### Conclusion

Our findings confirmed that SA prevalence among LBC was higher than among NLBC in rural China. Neglect, physical abuse, negative life events, loneliness, and parenting style were risk factors for SAs while more communication with LBC by guardians may reduce the burden of suicide in this group. However, further research is needed to explain how risk factors play a role at both the psychological, social, and family background levels, which would benefit suicide intervention and prevention policies in rural China and worldwide. SAs present an important public health issue in rural China, and more attention should be paid to suicide prevention among students from this area, especially those who have been left behind.

## Supporting information

S1 Table Raw data of a comparative analysis of suicide attempts in left-behind children and non-left-behind children in rural China analysis. Data are from the nationwide study on mental health outcomes among adolescents in rural China whose authors may be contacted at the Department of Child, Adolescence and Woman Health Care, School of Public Health, Tongji Medical College, Huazhong University of Science and Technology, 430030,Wuhan, Peoples Republic of China.
(XLSX)
